# Chemoprotection and Immunostimulation of Alpha-Lipoic Acid Against Chlorpyrifos-Induced Toxicosis in Nile Tilapia, *Oreochromis niloticus*

**DOI:** 10.1155/sci5/9985059

**Published:** 2025-07-09

**Authors:** Bilal A. Paray, Eijaz A. Bhat, Abdulkadir O. Adebayo, Femi J. Fawole, Ibrahim Adeshina

**Affiliations:** ^1^Department of Zoology, College of Science, King Saud University, P.O. Box 2455, Riyadh 11451, Saudi Arabia; ^2^Microbiology/Molecular Physiology of Prokaryotes, Institute of Biology II, University of Freiburg, Schänzlestraβe 1, Freiburg 79104, Germany; ^3^Department of Aquaculture and Fisheries, University of Ilorin, Ilorin, Nigeria; ^4^School of Aquaculture, National University of Agriculture, Porto-Novo, Benin

**Keywords:** antioxidant, feed supplement, immunity, intoxicant, Nile tilapia

## Abstract

This paper examined the chemoprotective and immunomodulatory effects of alpha-lipoic acid (ALA) against chlorpyrifos (CPL)-induced toxicosis in Nile tilapia, *Oreochromis niloticus*. Fish (*n* = 120, mean weight = 8.8 ± 0.6 g) were divided into 12 tanks (100-L capacity, 10 fish/tank) to represent two groups. Group 1 (60 fish) was fed a diet (300 g/kg crude protein) containing 40 mg ALA/kg feed, while Group 2 (60 fish) was fed a basal diet without ALA. Then, 30 fish from each group were exposed to 450 μg CPL/L. Thus, the experimental treatments were control, CPL (basal diet and exposed to CPL), ALA (ALA-based diet), and ALA + CPL (ALA-based and exposed to CPL) for 14 days. Fish fed with ALA alone showed significantly (*p*=0.001) higher survival rates (100%), improved hematological and biochemical profiles, enhanced antioxidant activity, and stronger immune responses than CPL-exposed fish alone. CPL exposure caused severe physiological disorders and histological damage, particularly in the liver. However, ALA + CPL interactions mitigated these adverse effects, restoring tissue integrity and functionality. The findings underscore ALA's potential as a dietary intervention to reduce environmental toxicant-induced stress in aquaculture, improving fish health and resilience against toxins.

## 1. Introduction

Aquaculture is one of the fastest-expanding subsectors, and it has become a major business as a result of a massive decline in supply from captured fisheries, as well as an increase in population [1]. Aquaculture, with an all-time record of 130.9 million tons, currently surpassed production from captured fisheries (91 million tons) [2]. Around the world, clay ponds are the most common type of pond [2] where aquaculture activities are often conducted. This is due to the fact that earthen ponds are more manageable, offer extra natural food, and boost fish productivity. Nile tilapia, *Oreochromis niloticus*, is one of the most popular fish species raised in earthen ponds. It accepts formulated feed and yields appreciable growth with a high economic value [3, 4]. Currently, tilapia production represents about 8% of total finfish production [2], and it is likely to increase in the coming years. This success can be associated with the improved and intensive farming techniques being deployed. However, toxins and pollutants, particularly those from insecticides, herbicides, and preservatives used in agriculture, have a negative impact on tilapia production and aquaculture in general [5, 6]. This is because a lot of crop farming is done close to rivers, dams, and other sources of water for aquaculture; thus, chemicals such as chlorpyrifos (CPL) used in farming end up in these waters through runoff and seepage [7].

CPL is an organophosphate pesticide commonly used to destroy pests, insects, and crops due to its ability to obstruct insect nervous systems by preventing the acetylcholinesterase (AChE) enzyme from functioning [8]. Additionally, studies have demonstrated that toxic mechanisms in fish exposed to insecticides and pesticides cause oxidative stress and mortalities [9–11]. The primary mechanism of CPL toxicity in fish involves the inhibition of AChE, an essential enzyme responsible for breaking down the neurotransmitter acetylcholine in synaptic clefts, thus leading to the accumulation of acetylcholine at nerve endings and cholinergic overstimulation. This causes neuromuscular dysfunction, manifested by symptoms such as abnormal swimming behavior, convulsions, paralysis, and eventually death in severe cases [10]. Furthermore, CPL generates oxidative stress by increasing the production of reactive oxygen species (ROS), which disrupt cellular antioxidant defenses. This oxidative damage affects vital organs, including the liver, kidney, and gills, leading to impaired metabolism, detoxification processes, and osmoregulation [10, 11]. Banded gourami, *Trichogaster fasciata*, exposed to 500 μg CPL/L, resulted in 100% mortality within 15 days, while 50 μg CPL/L greatly altered its reproductive tissue over 60 days [12]. In another study, 17% mortality of mosquitofish (*Gambusia affinis*) was reported due to exposure to 67 μg CPL/L [13]. Therefore, the need to derive a natural antioxidant such as alpha-lipoic acid (ALA) to mitigate the effects of CPL cannot be overemphasized.

ALA is a natural compound that stimulates protection against cellular damage and enhances carbohydrate hydrolysis for easier energy production for all physiological functions [14, 15]. Studies have demonstrated how ALA has been deployed in a wide field of study, including aquaculture, due to its antioxidant and protective properties [16–19]. Additionally, the usage of ALA affects the production of glutamate cysteine ligase (GCL) and glutathione-S-transferase (GST), which enhances cells' and organs' capacity to detoxify and act as antioxidants [16, 20]. Aquaculture could benefit from using ALA as a good preventive measure because it can cross the blood barriers and protect against foreign invasions and toxins [18, 21]. Therefore, it seems that ALA could act as an immunomodulatory and chemopreventive agent in fish against toxicosis [10]. Its application in preventing CPL-induced toxicosis in Nile tilapia has not been thoroughly explained. Thus, chemoprotective and immunostimulatory effects of ALA against CPL-induced toxicosis in Nile tilapia were investigated in this study.

## 2. Materials and Methods

### 2.1. Preparation and Exposure to CPL Intoxicant

A commercial CPL solution (CPL 20% EC: chlorpyriphos a.i. 20.00 w/w; inert ingredients 80.00 w/w) was purchased from Shandong Weifang Rainbow Chemical Co., Ltd., Shandong, China. The LD_50_ of CPL solution was determined using the Miller and Tainter method [19]. In brief, fish (mean weight = 8.0 g) were exposed to the intoxicant. Fish were not fed, and the water was not changed for the 96 h of the experiment. The lowest tolerated dose that gave 100% mortality and the highest dose that gave 0% mortality by the hit-and-trial method were determined. After that, five doses between these doses were selected, and mortality was recorded as a result of the doses. The correction factor to 0% and 100% mortality groups was calculated for 0% dead = 100 (0.25/*n*) and for 100% dead = 100 (*n* − 0.25/*n*), where *n* is the number of mortalities. Furthermore, probit units were read after converting mortality values to probit values, plotted against log doses, and read the LD50 value (450 μg/L) as the dose that corresponds to Probit 5.

### 2.2. Preparation of ALA Diets and Administration

The ALA was obtained from 21st Century HealthCare, Inc., 2119 S. Wilson St., Temple, AZ, 85,282, USA. The ALA dosage used in this study was determined based on the research by Amado et al. [5], who found that 40-mg ALA/kg feed is the optimal amount to induce the expression of the GST isoform, increase antioxidant profiles, and start the detoxification process in grass carp, *Ctenopharyngodon idella*. Two isonitrogenous diets (30% crude protein, CP) were prepared where ALA levels (0.0 and 0.04 g/kg feed) were mixed with soybean oil firstly and added to other ingredients with adequate amount of warm water to produce stiff dough (Table 1). Thereafter, the dough was pelleted in a hand-pelleting machine through a 2.0-mm pellet disc (Model HSDGP-60, Zhengzhou E.P. Machinery, Zhengzhou, China). The dried diets were kept at −20°C until use. The proximate chemical composition of the experimental diets (Table 1) was determined according to AOAC (2005) where dry matter (AOAC-930.15), crude protein (AOAC-960.52), ether extract (AOAC-945.38 F), ash contents (AOAC-923.03), and crude fiber (AOAC 962.09) were measured using standard methods. Metabolizable energy was calculated according to NRC [22] as follows: metabolizable energy = (5.67 × protein [g]) + (9.45 × lipid [g]) + (4.11 × nitrogen-free extract [g] + fiber [g]), where protein = 5.65 kcal/g, lipid = 9.45 kcal/g, and carbohydrate = 4.11 kcal/g). The value was then expressed as MJ/kg (i.e., 1 kcal = 1 × 0.004184).

### 2.3. Fish Culture

Juveniles of Nile tilapia, *O. niloticus* (*n* = 120, mean weight = 8.8 ± 0.6 g), were acclimatized for 14 days and fed with the commercial feed (30% CP) thrice a day (07:00 h, 12:00 h, and 17:00 h) up to apparent satiation. Then, the fish were distributed into 12 tanks (100-L capacity) at a stocking density of 10 fish per tank to represent four treatments with three replicates for each. Fish were divided into 12 tanks (100-L capacity, 10 fish/tank) to represent two groups. Group 1 (60 fish) was fed with a diet (300 g/kg crude protein) containing 40 mg ALA/kg feed, while Group 2 (60 fish) was fed with a basal diet without wet ALA. Then, 30 fish from each group were exposed to 450 μg CPL/L. Thus, the experimental treatments were control, CPL (basal diet and exposed to CPL), ALA (ALA-based diet), and ALA + CPL (ALA-based and exposed to CPL) for 14 days. Fish in the CPL-exposed group were exposed to 450 μg CPL/L for 24 h before administration of treatments. The exposure concentration was analytically confirmed using gas chromatography (Agilent 7890A, Avondale, PA, USA) and mass spectrometry (Agilent 6975, Avondale, PA, USA) (GC/MS) equipped with the HP column of 5 m long (0.25 m in diameter and 0.25 cm internal diameter) Agilent 190,915-433 HP-5M and compared to standard reference materials and certified control samples. The samples were replicated thrice to ensure precision. The fish tanks were connected with air-pumps containing air stones. The water temperature, dissolved oxygen, and pH degree were monitored two times per day with the aid of automatic multiparameters' probe (Hanna HI-9147, HACH Co., Loveland, Co., USA) throughout the 14 days of the experiment, and their values ranged between 27.2°C and 29.8°C, 6.1 and 6.9 mg/L, and 7.98 and 8.39, respectively. All these parameters were within the recommended values for rearing of fish [23].

### 2.4. Evaluation of Survival Rate of Fish

Survival rate and the level of protection were estimated as survival rate (%) = number of survived fish/initial number of fish × 100 [1, 5].

### 2.5. Assessment of Hematobiochemical Profiles

Sodium bicarbonate–buffered tricaine methane sulfonate (MS222, Sigma-Aldrich, USA; 30 mg/L) was used to anesthetize two fish from each tank. Then, a syringe and needle were used to draw blood from the caudal veins. After that, the blood was split into two parts. The first component was stored in lithium heparin-containing anticoagulant vials to evaluate hematological parameters. Briefly, packed cell volume (PCV, %), red blood cells (RBC, × 10^6^/μL), white blood cells (WBC, × 10^3^/μL), and platelets (Plat, × 10^6^/μL) were examined using Brown's method [18, 24, 25], while hemoglobin (Hb, g/dL) was determined using the method of Vankampen and Ziglstra [26]. The Wright–Giemsa stain method was used with a light microscope (Olympus CX21, Japan, 1000 magnification) to estimate differential counts of lymphocytes (Lym, %), heterocytes (Het, %), monocytes (Mon, %), eosinophils (Eos, %), and basophils (Bas, %).

The second portion of the blood was centrifuged (5000 × g) for 15 min at room temperature after being allowed to clot at room temperature. Then, the concentrations of urea and creatinine were determined using the techniques developed by Lausen [27] and Coulombe and Favreau [28], respectively. The difference between total protein (TP, g/dL) and albumin (ALB, g/dL) was used to calculate the amount of globulin [29]. According to the Reitman and Frankel [29] method, the aspartate (AST, IU/L) and alanine aminotransferase (ALT, IU/L) activities were measured colorimetrically [30].

### 2.6. Determination of Antioxidant and Immune Parameters

Euthanasia was carried out following the two-step procedure of the American Veterinary Medical Association [31] via immersion in an MS-222 solution (1000 mg/L water) and the secondary penetrating captive bolt method. The fish's liver, kidney, and brain samples were aseptically collected and homogenized according to the method of Amado et al. [5]. In brief, the samples were homogenized in a solution of Tris-HCl, EDTA, and Mg^2+^ at concentrations of 100 mM, 2 mM, and 5 mM (1:5—w/v), respectively. The samples were then centrifuged at 10,000 × *g* for 20 min at 4°C to test the superoxide dismutase (SOD, Cat. No.: SD124) [32], catalase (CAT) [33], and glutathione peroxidase (GPx, Cat. No.: RS504) [34] using diagnostic reagent kits from Randox Laboratories in Crumlin, County, Antrim, UK [35]. The activities of reduced glutathione (GSH) [36], GCL [37], and GST [38] were evaluated after the conjugation of 1-mM glutathione and 1-mM 1-chloro-2,4-dinitrobenzene (CDNB) at 340 nm. Agar wells were made on a round reaction plate, and each well received aliquot (25 L) of the GCL reaction cocktail. The reaction plate was pipetted with the aliquots, then 25 L of previously dissolved 2-mM cysteine in a buffer [39, 40].

In addition, serum lysozyme, respiratory burst activities (RBAs), and TP were determined using the turbidimetric method [41], the nitroblue tetrazolium dye method [42], and the colorimetric method [33], respectively. Briefly, 0.60 mg/mL of *Micrococcus luteus* was cast in 1% agarose gel (Difco BD Co, Franklin Lakes, NJ) with 50-mM phosphate buffer (pH 6.2). Wells (6 mm) were created on nutrient agar plates and were filled with 25 μL of serum samples and incubated for 20 h at 25°C. Lysozyme activity was calculated from a standard curve prepared with lysozyme from chicken egg white. In addition, RBA was measured using the BOC redox kit (Cat. No.: DHR-123, BOC Redox Technologies, Vivero Ciencias de la Salud C. Colegio Santo Domingo de Guzmán – 33,011 Oviedo Asturias – Spain). Briefly, 10 μL of each sample was added to a tube containing 10 μL of DHR-123 and incubated at 37°C for 15 min, after which the solution was added to 25 μL of phorbol 12-myristate 13-acetate/phosphate-buffered saline and returned to the incubator at 37°C for 45 min. Then, 2 mL of RBC lysis buffer was added to the solution and incubated at 37°C for 20 min before being centrifuged at 500 × *g* at RT. The supernatants were removed before the cells were resuspended in 0.5-mL assay buffer. The RBA was then measured by flow cytometry with a fluorescence emission at 530 nm [43, 44].

### 2.7. Histopathological Evaluation of the Liver, Kidney, and Brain

The livers, kidneys, and brain tissues were fixed in Bouin's fluid for 24 h and then transferred into 20% neutral buffered formalin (Sigma-Aldrich, St. Louis, MO). The samples were then washed thoroughly under tap water and dehydrated through ascending grades of ethanol, cleaned in chloroform, and routinely processed into paraffin wax at 60°C for histology, sectioned at 5 μm, and stained with hematoxylin and eosin (Sigma-Aldrich) [45].

### 2.8. Statistical Analysis

Prior to the analysis, data were subjected to normality of distribution and homogeneity of variance tests using Shapiro–Wilk's Levene and Kolmogorov–Smirnov tests. Then, the data were analyzed using two-way analysis of variance to explore the effects of ALA, CPL, and their interaction for survival rate, relative protection, antioxidant activities, and immune responses of Nile tilapia against CPL-induced toxicosis, and means were separated using Tukey's test at the significance level of *p* < 0.05 with the aid of the SPSS Version 20.0 [46, 47].

## 3. Results

**Figure 1:**
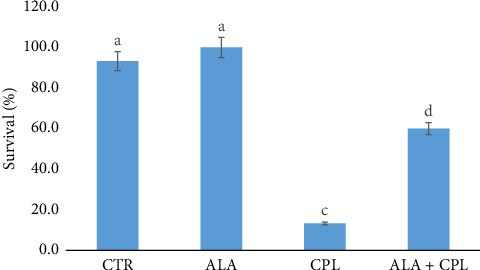
Survival and relative level of protection of Nile tilapia fed on ALA and chlorpyrifos (CPL)-induced toxicity. CTR, fish-fed diet without ALA and CPL exposure; ALA, fish-fed diet enriched with alpha-lipoic acid (ALA) only; CPL, fish exposed to chlorpyrifos (CPL); ALA + CPL, fish fed with ALA and exposed to CPL.

Figure 1 shows the survival rate of Nile tilapia in the treated groups. The outcome revealed that survival rates among the groups varied significantly (*p*=0.001). The least survival rate was recorded in the group exposed to CPL (13.3%), while those in the ALA group had the highest survival rate (100.0%), an indication of the toxicity of CPL in Nile tilapia. The hematological profiles of Nile tilapia in this study are shown in Table 2. When the ALA-based diet was fed to fish, the hematological indices were noticeably higher in comparison to the control or CPL groups (*p*=0.001). Specifically, PCV, Hb, RBCs, WBCs, and Lym values were higher in fish-fed ALA, while their corresponding lowest values were obtained in the CPL group (Table 2). The interaction between the ALA and CPL was also significant (*p*=0.001). Also, Het, Mon, Eos, and Bas levels were significantly (*p*=0.002; *p*=0.013, respectively) higher in the CPL group than in the CTR group. Moreover, the Plat value showed significant reduction in the fish exposed to CPL (Table 2).

Liver and kidney function indices of Nile tilapia fed with ALA and exposed to CPL are presented in Table 3. There were significant differences in the biochemical profiles (*p* < 0.05) of the fish across the groups. There were significantly higher AST (*p*=0.001), ALT (*p*=0.001), urea (*p*=0.001), and creatinine (*p*=0.001) values in the CPL group when compared to the CTR group (Table 3). Notably, the interaction between ALA and CPL significantly influence the level of AST (*p*=0.001), urea (*p*=0.003), and creatinine values but did not affect the AST ((*p*=0.546) level in Nile tilapia, indicating the protective effect of ALA against CPL-induced toxicity.  Table 4 depicts the innate immunity of Nile tilapia in the treatment groups. ALB, globulin, TP, LYZ, and RBA were significantly (*p*=0.001) elevated in Nile tilapia–fed dietary-ALA compared to the control group (Table 4). However, the exposure to CPL significantly reduces ALB, globulin, TP, LYZ, and RBA levels in Nile tilapia (Table 4).

Changes in antioxidant index levels are presented in Table 5. SOD activity varied significantly across treatments in all tissues. CPL exposure significantly (*p*=0.001) reduced SOD activity, particularly in the kidney and brain compared to the control group. ALA supplementation restored SOD activity, with the ALA + CPL group showing levels comparable to the control in the liver, kidney, and brain. The effects were significant for ALA, CPL, and their interaction (*p*=0.001; Table 5). However, CPL exposure led to a significant (*p*=0.001) reduction in CAT activity, particularly in the kidney. ALA supplementation alone maintained or slightly increased CAT levels, and the ALA + CPL group restored levels close to the control in the liver and kidney. In the brain, CAT activity showed no significant change in ALA, CPL, and their interaction (*p* > 0.05). GPX activity significantly decreased with CPL exposure in the liver (*p*=0.01; Table 5). The ALA + CPL group partially restored GPX activity in the liver but showed no significant changes in the kidney and brain (Table 5). GST activity was markedly reduced in the CPL group across all organs (*p*=0.001). ALA supplementation significantly elevated GST levels, with the ALA + CPL group, showing partial restoration in all tissues (*p*=0.001) (Table 5). CPL exposure significantly depleted GSH levels in the liver, kidney, and brain (*p*=0.001). ALA alone increased GSH levels significantly in all tissues, and the ALA + CPL group showed significant amelioration compared to CPL alone. CPL exposure significantly reduced GCL activity in the kidney and brain (*p*=0.001), while the liver showed no significant (*p*=0.034) change. The ALA + CPL group restored GCL activity close to control levels in the kidney and brain (Table 5).

**Figure 2:**
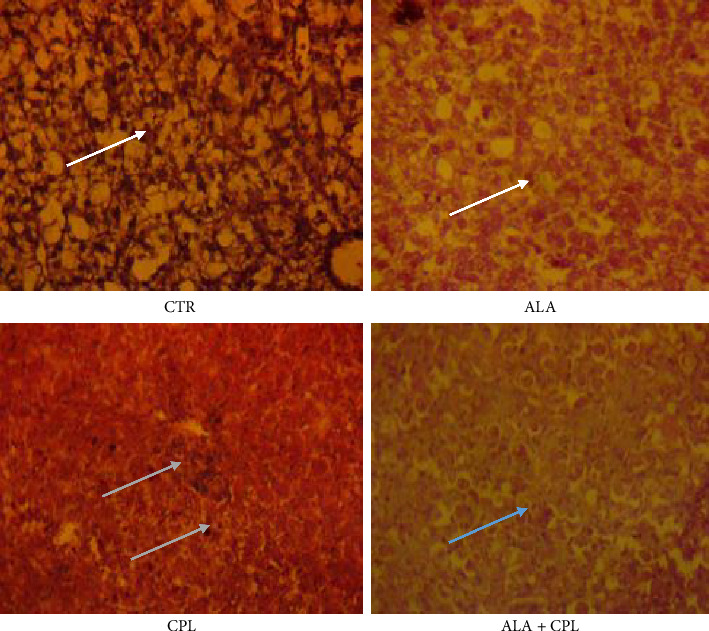
Histological changes in the liver of Nile tilapia fed on ALA-based diet against CPL-induced toxicosis (staining: hematoxylin and eosin (H&E); magnification: 400×. Scale bar = 50 μm). Note: CTR = fish with no ALA and no CPL; ALA = fish fed with alpha-lipoic acid only; CPL = fish exposed to chlorpyrifos; ALA + CPL = fish fed with alpha-lipoic acid and exposed to chlorpyrifos. Arrows: arrow (white) = no visible lesion normal hepatocytes in the liver; arrow (green) = centrilobular vacuolation, patchy hepatocyte necrosis, mononuclear cellular infiltrates, and hepatocellular necrosis; arrow (blue) = diffuse hepatocellular degeneration and mild necrosis.

**Figure 3:**
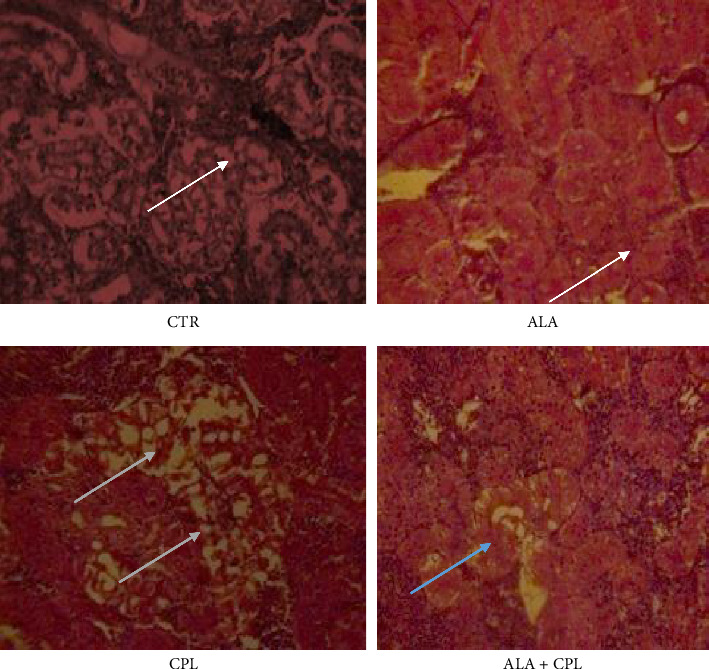
Histological changes in the kidney of Nile tilapia fed on ALA-based diet against CPL-induced toxicity (staining: hematoxylin and eosin (H&E); magnification: 400×. Scale bar = 50 μm.). Note: CTR = fish with no ALA and no CPL, ALA = fish fed with alpha-lipoic acid only, CPL = fish exposed to chlorpyrifos, and ALA + CPL = fish fed with alpha-lipoic acid and exposed to chlorpyrifos. Arrows: arrow (white) = no visible alteration; arrow (green) = mild clogging and tubular epithelium degeneration and necrosis; arrow (blue) = mild clogging.

**Figure 4:**
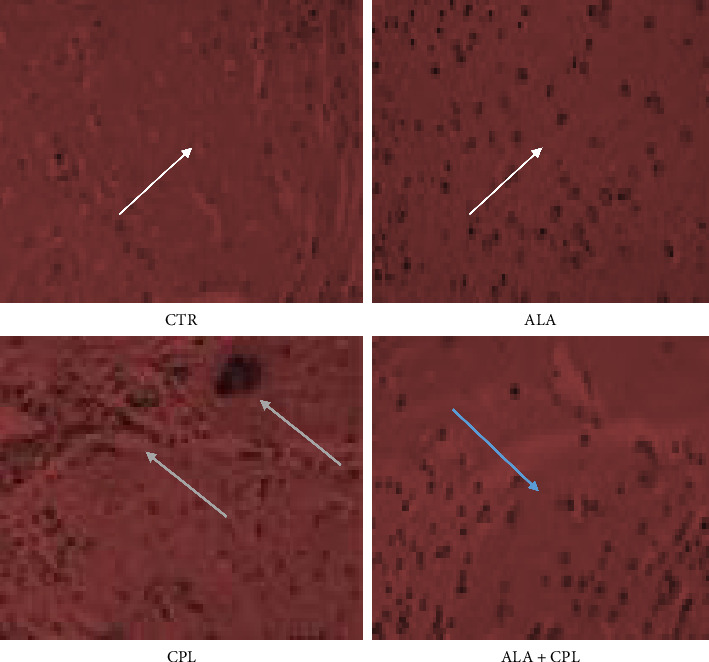
Histological changes in the brain of Nile tilapia fed on ALA-based diet against CPL-induced toxicity (staining: hematoxylin and eosin (H&E); magnification: 400×. Scale bar = 50 μm.). Note: CTR = fish with no ALA and no CPL, ALA = fish fed with alpha-lipoic acid only, CPL = fish exposed to chlorpyrifos, and ALA + CPL = fish fed with alpha-lipoic acid and exposed to chlorpyrifos. Arrows: arrow (white) = no observable lesion; arrow (green) = high levels of apoptosis, cell inflammation, choroidal plexus degeneration and aggregation of rodlet cells; arrow (blue) = low levels of apoptosis.

The histological alterations in the liver, kidney, and brain tissues of Nile tilapia fed with ALA to prevent toxicosis caused by CPL exposure are shown in Figures 2, 3, and 4, respectively. In contrast to fish fed with ALA and the control, the liver of fish exposed to CPL intoxicant only showed normal hepatocytes in the liver along with sporadic cytoplasmic vacuolation, centrilobular vacuolation, patchy hepatocyte necrosis, mononuclear cellular infiltrates, and diffuse hepatocellular degeneration and necrosis (Figure 2). Furthermore, in the ALA + CPL group, the integrity of the liver was restored.  Figure 3 revealed that supplementation of ALA in Nile tilapia did not cause any histological changes in the kidney. Instead, there was mild clogging, and tubular epithelium degeneration and necrosis in the kidney of the fish in the CPL group (Figure 3). In addition, Figure 4 shows that fish brains were highly marked with high levels of apoptosis, cell inflammation in the CPL group when compared to the control group, which did not show any observable lesion.

## 4. Discussion

The survival rate data indicated that CPL is harmful to Nile tilapia; however, fortifying the fish with ALA supplementation significantly improved their survival rates. This demonstrates how ALA protects fish from toxicosis brought on by CPL exposure. According to Kütter et al. [41] and Dong-Liang et al. [42], Plata pompano, *Trachinotus marginatus*, and Nile tilapia fed on dietary ALA showed higher survival rates than the control. The toxicity of CPL observed in this study emphasizes the harmful effects of chemical pollution on fish survival. In zebrafish, *Danio rerio*, exposed to 100 ng/mL of CPL, mortality was 58.3% [48], while 100% mortality was recorded in African catfish, *Clarias gariepinus*, and Nile tilapia due to the exposure to 24 mg/L CPL [49]. Moreover, the high survival rate observed in the ALA group (100.0%) aligns with Behairy et al. [50], who attributed the high survival rate (100%) in tilapia supplemented with ALA to its antioxidant properties and its ability to mitigate cellular damage [50]. The clear difference between the survival rates of the CPL and ALA groups further established the protective role of ALA in fish, particularly in mitigating the effects of environmental toxins.

Hematological profiles are frequently used and have proven to be useful in examining fish health [33, 51, 52]. Blood profiles measured in the current study, including PCV, Hb, RBCs, WBCs, and Plat, were significantly decreased as a result of exposure to CPL, but these aberrations were reversed in fish fed with ALA and as shown with their interaction. These findings also showed that dietary ALA stimulated the Nile tilapia's hematological function, which in turn promoted oxygenation in the fish tissue as indicated by the PCV, RBC, and Hb levels. The numbers of hematopoietic cells, RBCs, which carry oxygen, and WBCs, which are leukocytes that fight infections, indicate that fish fortified with ALA had superior immunity, which is why a higher survival rate was observed in this study. Additionally, lower levels of RBC and Hb in the CPL group suggest increased erythrocyte breakdown and/or reduced RBC and Hb synthesis. Usually, when fish are exposed to toxins, the leucopoietic and lymphopoiesis processes are stimulated, releasing lymphomyeloid and producing WBCs as a result. In contrast, higher Het, Mon, Eos, and Bas in fish from the CPL group suggest that the immune system of Nile tilapia is understressed. Het, Mon, Bas, and Eos are useful for eliminating invading infections and chemicals as well as for triggering an inflammatory response [53]. The body of the fish is stimulated by the CPL intoxicant, which is what causes the elevated amounts of Het, Mon, Eos, and Bas [54].

Furthermore, the elevated levels of AST, ALT, urea, and creatine in the CPL group buttress the hepatotoxic and nephrotoxic effects of CPL, which indicate liver damage and result in the release of these enzymes into the blood stream [55]. Particularly, liver aminotransferases that reflect the health and functionality of the liver are AST and ALT. The decrease in AST and ALT levels in fish treated with ALA and ALA + CPL suggests that the liver cell membrane integrity is being protected against stresses. This decrease in enzyme activity demonstrates that the membrane stability against free radical–mediated toxins was improved by phytase-evoked protection against hepatic damage. The outcomes proved that the fish were not under any stress. Similarly, increased urea and creatinine levels are hallmark indicators of renal dysfunction, suggesting compromised excretory functions due to CPL exposure. These findings are corroborated by previous studies reporting oxidative stress and organ damage in fish exposed to CPL [56, 57].

A typical fish's urea and creatinine levels, according to NKF [58], should be between 7 and 20 mg/dL and 0.54 and 0.72 mg/dL, respectively. In this study, fish exposed to a CPL had creatine levels higher than the recommended levels, but due to ALA's protective effects on fish, this value was redeemed in the ALA + CPL group. The body's level of nonprotein nitrogen and the kidneys' and livers' capacity to get rid of metabolic waste are both indicators of how well-functioning the kidney and liver are [58]. The results of this study differ from those published by Agbede et al. [59] and Oyelese et al. [60], but they are comparable to those of Ajeniyi and Solomon [61] and Adeshina et al. [51]. Urea, creatinine, and TP levels were simultaneously reduced in grass carp [62] and *Labeo bata* [63] exposed to CPL, respectively. The higher creatinine levels in the CPL group suggest a urinary tract blockage and inadequate blood supply to the kidney caused by CPL.

Fish immunity is a crucial defense mechanism that guards against and shields fish from foreign invasion. It was shown in this study that fish fed with ALA and exposed to CPL exhibited wide alterations in their immune indices. The elevated values of ALB, globulin, TP, and LYZ and RBA in the ALA-fed group pointed to ALA's influence on protein metabolism in the fish. Zhang et al. [64] and Rahman et al. [65] documented similar observations that dietary ALA enhances fish immunity and metabolic activity through its anti-inflammatory and antioxidative properties. Contrariwise, fish in the CPL group expressed great reduction in ALB, globulin, total, and protein levels when compared to the CTR group. These findings are similar to the observations of El-Demerdash et al. [66] and Ali et al. [67], who noted the hepatotoxic effects of CPL on fish resulting in compromised liver functionality and protein synthesis. Similarly, Ghayyur et al. [62] reported a decreased TP in *Oreochromis mossambicus* fish exposed to 1–100 μg/L CPL. Furthermore, LYZ is a cationic protein that is present in mucus, lymphoid tissue, and plasma. LYZ is produced in the liver of fish and is used in a variety of defense processes, such as bacteriolysis, opsonization, and immunological response, among others [68]. One of the most significant cellular defense mechanisms in fish is respiratory burst which is crucial in neutralizing harmful and pathogenic organisms inside the cell. Production of ROS production during a respiratory burst stimulates the phagocytic cell membrane, activating the NADPH oxidase that is linked to the membrane and causes an increase in oxygen consumption/ROS which are known to be toxic for bacterial pathogens [43, 69]. Fish with increased phagocytic activity and immune systems may also have increased RBA [56, 57, 68]. The findings of this study are consistent with those of Ghayyur et al. [62], who found that Nile tilapia fed on a diet fortified with ALA had greater lysozyme and RBA. The outcome of this study reiterates ALA's immunomodulatory functions in fish.

The antioxidant parameters further highlight the detrimental effects of CPL and the mitigating effects of ALA. CAT and SOD are enzymes found in a variety of animals, including fish. These enzymes have important functions in defending the cell from the harmful effects of toxins [70]. SOD catalyzes the dismutation of the superoxide ion (O2-) to hydrogen peroxide and oxygen molecules in the oxidative energy process, which subsequently moderates the harmful oxidative processes in cells. According to Vazquez and Nostro [71], SOD, CAT, and GPx activities have developed into reliable biomarkers for toxic substances and fish health. CAT is a further significant antioxidant enzyme that is present in almost all aerobic organisms and converts two molecules of hydrogen peroxide into one molecule of oxygen and two molecules of water in a two-step reaction. CAT deficiency is a sign of damage brought on by illnesses and toxins, while converting lipid hydroperoxides to alcohols and free hydrogen peroxide to water, GPx, an essential enzyme with peroxidase activity, protects the organism against oxidative damage [71].

Fish in the CPL group showed a great reduction in SOD, CAT, GPS, GSH, GCL, and GST levels, signifying oxidative stress. In a similar study by Kavitha and Rao [72], it was reported that mosquito fish, *G. affinis,* exposed to 297 μg CPL/L, had diminished antioxidant enzymes. Remarkably, ALA supplementation significantly improved antioxidant enzyme activities in Nile tilapia, especially in the ALA + CPL group, because ALA is capable of inducing the regeneration of an endogenous antioxidant [5]. The higher antioxidant enzyme activities observed in this study are consistent with the reports by Bhattacharya et al. [73], who demonstrated that dietary ALA improved oxidative stress markers in fish exposed to organophosphate pesticides. Any substance that might activate the expression of GST could boost defenses against CPL-induced toxicity because intoxicants, including pesticide exposure, can alter GST expression [5]. In this study, CPL exposure reduced the GST activities in Nile tilapia, whereas ALA-CPL fish showed elevated GST levels in the liver, kidney, and brain, reaching levels that were roughly equivalent to those of the ALA and control groups. The ALA + CPL interactions were most expressed in the liver. This is unsurprising, given that the liver is the primary site for detoxification and is thus highly susceptible to oxidative damage from toxicants such as CPL. In Nile tilapia, CPL toxicity induced oxidative stress and biochemical disruptions [74–76], while Bhattacharya et al. [73] highlighted the role of ALA as an antioxidant agent in mitigating pesticide toxicity and improving both biochemical and antioxidant profiles in Nile tilapia.

The exposure of Nile tilapia to CPL revealed significant histopathological alterations in the liver, kidney, and brain tissues; however, these effects were greatly ameliorated in the ALA + CPL group. These findings illustrate the role of ALA in preventing and/or regulating oxidative and inflammatory effects in the tissue integrity. These alterations induced by CPL suggest that the fish's liver was not able to properly carry out the detoxification and metabolic functions. However, restoration of these hepatic changes was greatly expressed in the ALA + CPL group and further evident by the lack of significant histological alterations. Sakamoto et al. [77] reported that ALA supplementation greatly ameliorated the hepatic lipid peroxidation in common carp exposed to pesticides.

In the kidney, CPL exposure led to mild clogging, tubular epithelial degeneration, and necrosis, which agreed with Zhao et al. [78], who reported that CPL has nephrotoxic effects such as tubular damage and glomerular congestion in common carp. On the other hand, the absence of alteration in renal tissues in the ALA + CPL group suggests that ALA inhibited oxidative stress and inflammation in renal tissues. These findings are in accordance with Wang et al. [79], who documented that dietary ALA prevented renal dysfunction in tilapia exposed to heavy metals.

In addition, the brain tissues of fish in the CPL group had apoptosis, choroidal plexus degeneration, and cell inflammation, which suggests the neurotoxic effects of CPL. In common carp, organophosphate pesticide caused neurotoxicity in the fish's brain [80]. Interestingly, the ALA + CPL group displayed a huge reduction in brain lesions establishing the protective effect of ALA in modulating neuroinflammation as reported in zebrafish exposed to 500 μg CPL/L [81]. Mostly, the previous studies predominantly focused on the biochemical and molecular effects of ALA; this study detailed the histological evidence, showing the potentiality of ALA in organ maintenance and tissue integrity.

## 5. Conclusion

This study demonstrates the protective effects of dietary ALA against CPL-induced toxicity in Nile tilapia. Fish fed with ALA-supplemented diet exhibited better survival, enhanced hematological and biochemical profiles, improved antioxidant capacity, and strengthened immune response post CPL exposure. However, ALA + CPL interaction greatly alleviated such pathological alterations by restoring tissue integrity. Therefore, ALA supplementation shows great potential as a dietary intervention to combat environmental toxicant-induced stress in aquaculture.

## Figures and Tables

**Table 1:** Ingredients and proximate composition (g/kg diet on dry matter basis) of the basal and alpha-lipoic acid (ALA)–based diets.

Ingredients	ALA levels	
	0.0 (control)	0.04 g/kg diet
Fish meal (550 g/kg crude protein)	250	250
Soybean meal (450 g/kg crude protein)	250	250
Maize flour	430	429.96
Soybean oil	10	10
Vitamins premix^1^	20	20
Minerals premix^2^	20	20
Starch	20	20
ALA	0	0.04
Total	1000	1000
Metabolizable energy (MJ/kg diet)^3^	35.2	35.2
Proximate composition (g/kg)		
Dry matter	925	925
Crude protein	308	308
Nitrogen-free extract	79	79
Crude lipid	143	143
Total ash	81	81
Crude fiber	46	46

^1^Vitamins premix (per kg of premix): thiamine, 2.5 g; riboflavin, 2.5 g; pyridoxine, 2.0 g; inositol, 100.0 g; biotin, 0.3 g; pantothenic acid, 100.0 g; folic acid, 0.75 g; para-aminobenzoic acid, 2.5 g; vitamin C (coated), 900 mg, choline, 200.0 g; nicotinic acid, 10.0 g; cyanocobalamin, 0.005 g; α-tocopherol acetate, 20.1 g; menadione, 2.0 g; retinol palmitate, 100,000 IU; cholecalciferol, 500,000 IU.

^2^Minerals premix (per kg of premix): CaHPO_4_ 2H_2_O, 727.2 g; MgCO_3_ 7H_2_O, 127.5 g; KCl 50.0 g; NaCl, 60.0 g; FeC_6_H_5_O_7_ 3H_2_O, 25.0 g; ZnCO_3_, 5.5 g; MnCl_2_ 4H_2_O, 2.5 g; CuCl_2_, 0.785 g; CoCl_3_. 6H_2_O, 0.477 g; CaIO_3_ 6H_2_O, 0.295 g; CrCl_3_ 6H_2_O, 0.128 g; AlCl_3_ 6H_2_O, 0.54 g; Na_2_SeO_3_, 0.3 g.

^3^Metabolizable energy = (5.67 × 308 [g]) + (9.45 × 143 [g]) + (4.11 × 79 [g] + 46 [g]), where protein = 5.65 kcal/g; lipid = 9.45 kcal/g; carbohydrate = 4.11 kcal/g. The value was then expressed as MJ/kg (i.e., 1 kcal = 1 × 0.004184).

**Table 2:** Hematological profiles of Nile tilapia fed on alpha-lipoic acid against chlorpyrifos (CPL)-induced toxicity for 14 days.

Parameters	CTR	ALA	CPL	ALA + CPL	SEM	*p* values		
						ALA	CPL	ALA × CPL
Packed cell volumes (%)	28.76^b^	38.43^a^	16.32^c^	35.21^a^	4.23	< 0.001	< 0.001	< 0.001
Hemoglobin (g/dL)	12.42^b^	16.72^a^	6.48^c^	14.34^ab^	2.15	< 0.001	< 0.001	< 0.001
Red blood cell (× 10^6^/μL)	3.62^b^	4.67^a^	1.42^c^	4.25^b^	0.85	< 0.001	< 0.001	< 0.001
White blood cell (× 10^3^/μL)	185.11^a^	193.23^a^	122.41^b^	190.16^a^	3.95	< 0.001	0.001	0.002
Platelets (× 10^6^/μL)	284.16^a^	296.33^a^	165.25^b^	295.14^a^	16.24	0.007	0.258	0.262
Lymphocytes (%)	59.66^a^	59.69^a^	54.12^b^	58.68^a^	1.01	< 0.001	0.029	0.129
Heterocytes (%)	32.12^b^	32.14^b^	35.25^a^	32.35^b^	4.43	< 0.001	0.002	0.014
Monocytes (%)	3.46^b^	3.47^b^	4.05^a^	3.86^a^	1.16	< 0.001	< 0.001	< 0.001
Eosinophils (%)	3.16^b^	3.19^b^	4.21^a^	3.28^b^	0.17	0.003	0.013	0.015
Basophils (%)	1.60^c^	1.51^c^	2.37^a^	1.83^b^	0.06	0.002	0.010	0.005

*Note:* CTR = fish with no ALA and no CPL, ALA = fish fed with alpha-lipoic acid only, CPL = fish exposed to chlorpyrifos, anf ALA + CPL = fish fed with alpha-lipoic acid and exposed to chlorpyrifos. Means with different superscripts in the same row are significantly different (*p* < 0.05).

**Table 3:** Liver and kidney function indices of Nile tilapia fed on alpha-lipoic acid against chlorpyrifos-induced toxicity for 14 days.

Variables	CTR	ALA	CPL	ALA + CPL	SEM	*p* value		
						ALA	CPL	ALA × CPL
AST (IU/L)	193.4^b^	180.7^c^	206.5^a^	191.6^b^	2.10	< 0.001	< 0.001	0.546
ALT (IU/L)	22.2^b^	19.1^c^	31.3^a^	22.4^b^	1.09	< 0.001	< 0.001	< 0.001
Urea (mg/dL)	6.98^b^	3.09^c^	9.32^a^	7.15^b^	0.683	< 0.001	< 0.001	0.003
Creatnine (mg/dL)	0.88^c^	0.42^b^	0.89^a^	0.85^a^	0.061	< 0.001	< 0.001	< 0.001

*Note:* CTR = fish with no ALA and no CPL, ALA = fish fed with alpha-lipoic acid only, CPL = fish exposed to chlorpyrifos, ALA + CPL = fish fed with alpha-lipoic acid and exposed to chlorpyrifos, AST = aspartate aminotransferase, and ALP = alanine aminotransferase. Means with different superscripts in the same row are significantly different (*p* < 0.05).

**Table 4:** Immune indices of Nile tilapia fed on alpha-lipoic acid against chlorpyrifos-induced toxicity for 14 days.

Variables	CTR	ALA	CPL	ALA + CPL	SEM	*p* value		
						ALA	CPL	ALA × CPL
Albumin (g/dL)	2.77^b^	3.68^a^	1.57^c^	3.58^a^	0.251	< 0.001	0.027	0.005
Globulin (g/dL)	1.45^b^	2.00^a^	1.00^c^	1.94^a^	0.117	0.001	0.017	0.837
Total protein (g/dL)	4.22^b^	5.68^a^	2.57^c^	5.52^a^	0.382	< 0.001	< 0.001	< 0.001
LYZ (U/mg protein)	3.16^b^	5.35^a^	1.17^d^	2.72^c^	0.450	< 0.001	< 0.001	< 0.001
RBA (U/mg/mL protein)	0.23^b^	0.43^a^	0.16^c^	0.21^b^	0.032	< 0.001	< 0.001	0.004

*Note:* CTR = fish with no ALA and no CPL, ALA = fish fed with alpha-lipoic acid only, CPL = fish exposed to chlorpyrifos, ALA + CPL = fish fed with alpha-lipoic acid and exposed to chlorpyrifos, and LYZ = lysozyme activity. Means with different superscripts in the same row are significantly different (*p* < 0.05).

Abbreviation: RBA, respiratory burst activity.

**Table 5:** Changes in antioxidant index levels in the liver, kidney, and brain of Nile tilapia fed on alpha-lipoic acid (ALA) against chlorpyrifos (CPL)-induced toxicity.

Parameters	Organs	CTR	ALA	CPL	ALA + CPL	SEM	*p* values		
							ALA	CPL	ALA × CPL
SOD (IU/mg protein)	Liver	20.44^b^	32.69^a^	15.91^c^	30.39^a^	2.089	< 0.001	< 0.001	0.007
	Kidney	52.47^b^	73.01^a^	23.00^c^	51.16^b^	5.212	< 0.001	< 0.001	< 0.001
	Brain	42.31^b^	51.46^a^	31.33^c^	45.07^b^	2.199	< 0.001	< 0.001	< 0.001

CAT (IU/mg protein)	Liver	18.15^b^	21.24^a^	11.31^c^	18.11^b^	1.06	< 0.001	< 0.001	< 0.001
	Kidney	35.26^a^	37.11^a^	14.52^c^	34.57^b^	1.03	< 0.001	< 0.001	< 0.001
	Brain	27.23^a^	28.23^a^	26.10^a^	26.24^a^	1.04	0.023	< 0.001	0.005

GPx (IU/mg protein)	Liver	89.25^b^	125.38^a^	42.17^c^	87.65^b^	2.56	< 0.001	< 0.001	< 0.001
	Kidney	1.51^a^	1.63^a^	1.48^a^	1.61^a^	0.12	0.012	0.659	0.713
	Brain	43.17^a^	44.05^a^	41.63^a^	41.89^a^	3.21	1.000	0.041	0.933

GST (IU/mg protein)	Liver	24.56^b^	28.14^a^	13.03^c^	25.52^b^	1.35	< 0.001	< 0.001	< 0.001
	Kidney	24.13^b^	26.25^a^	14.19^c^	28.33^a^	2.32	< 0.001	0.283	0.002
	Brain	37.35^b^	38.48^b^	25.33^c^	53.27^a^	1.06	< 0.001	< 0.001	< 0.001

GSH (nmol/mg protein)	Liver	84.32^b^	95.61^a^	43.07^c^	75.38^b^	4.27	< 0.001	< 0.001	< 0.001
	Kidney	96.42^b^	103.05^a^	39.47^c^	97.24^b^	3.91	< 0.001	< 0.001	< 0.001
	Brain	72.36^a^	82.49^a^	58.87^b^	71.45^a^	2.45	< 0.001	< 0.001	0.008

GCL (nmol/mg protein)	Liver	43.16^a^	44.23^a^	40.07^a^	42.78^a^	3.11	0.124	0.034	0.680
	Kidney	50.21^a^	57.16^a^	26.38^c^	48.69^b^	4.06	< 0.001	< 0.001	< 0.001
	Brain	42.17^b^	62.20^a^	21.34^c^	58.76^b^	3.18	< 0.001	< 0.001	0.004

*Note:* CTR = fish with no ALA and no CPL, ALA = fish fed with alpha-lipoic acid only, CPL = fish exposed to chlorpyrifos, ALA + CPL = fish fed with alpha-lipoic acid and exposed to chlorpyrifos, SOD = superoxide dismutase, CAT = catalase, GPX = glutathione peroxidase, and GSH = reduced glutathione. Means with different superscripts in the same row are significantly different (*p* < 0.05).

Abbreviations: GCL, glutamate cysteine ligase; GST, glutathione-S-transferase.

## Data Availability

The data that support the findings of this study are available from the corresponding author upon reasonable request.
